# The Role of STAT1 for Crosstalk between Fibroblasts and Colon Cancer Cells

**DOI:** 10.3389/fonc.2014.00088

**Published:** 2014-04-30

**Authors:** Pawan Kaler, Benjamin Y. Owusu, Leonard Augenlicht, Lidija Klampfer

**Affiliations:** ^1^Montefiore Medical Center, Bronx, NY, USA; ^2^Southern Research Institute, Birmingham, AL, USA; ^3^Albert Einstein Cancer Center, Bronx, NY, USA

**Keywords:** STAT1, colon cancer, tumor microenvironment, proliferation, fibroblasts

## Abstract

Signaling between tumor cells and the associated stroma has an important impact on cancer initiation and progression. The tumor microenvironment has a paradoxical role in tumor progression and fibroblasts, a major component of the tumor stroma, have been shown to either inhibit or promote cancer development. In this study, we established that normal intestinal fibroblasts activate STAT1 signaling in colon cancer cells and, in contrast to cancer-associated fibroblasts, inhibit growth of tumor cells. Treatment of 18Co fibroblasts with the proinflammatory cytokine TNFα interfered with their ability to trigger STAT1 signaling in cancer cells. Accordingly, intestinal myofibroblasts isolated from patients with ulcerative colitis or Crohn’s disease, which are activated and produce high levels of TNFα, failed to stimulate STAT1 signaling in tumor cells, demonstrating that activated myofibroblasts lose the ability to trigger growth-inhibitory STAT1 signaling in tumor cells. Finally, we confirmed that silencing of STAT1 in tumor cells alters the crosstalk between tumor cells and fibroblasts, suggesting STAT1 as a novel link between intestinal inflammation and colon cancer. We demonstrated that normal fibroblasts restrain the growth of carcinoma cells, at least in part, through the induction of STAT1 signaling in cancer cells and showed that changes in the microenvironment, as they occur in inflammatory bowel disease, alter the crosstalk between carcinoma cells and fibroblasts, perturb the homeostasis of intestinal tissue, and thereby contribute to tumor progression.

## Introduction

Progression of colon cancer relies on the communication of tumor cells with the adjacent stroma. Reciprocal interactions between tumor cells and stroma are mediated by soluble factors such as cytokines, growth factors, chemokines, proteases, and components of the extracellular matrix. Fibroblasts and myofibroblasts are the major stromal cell types associated with human carcinomas.

Cancer-associated fibroblasts (CAFs) have a role in both initiation and progression of cancer. The origin of CAFs and myofibroblasts is poorly understood. Experimental data suggest that myofibroblasts can be derived from epithelial cells via epithelial mesenchymal transition, they can differentiate from resident fibroblasts, adipocytes, or from mesenchymal or hematopoietic stem cells (1). Several tumor-derived factors, including TGFβ, have been shown to mediate the conversion of normal fibroblasts into CAFs (2). Tumor-associated fibroblasts are similar to fibroblasts found in wounded and fibrotic tissues. However, in contrast to wound healing, where activated fibroblasts revert to a quiescent phenotype once the process is completed, tumor-associated fibroblasts remain constitutively activated and thus aid in tumor growth and tumor survival. CAFs remain non-transformed, therefore genetic alterations are unlikely to contribute to their ability to drive tumor progression (3). They have been shown to support the survival and proliferation of cancer cell and to stimulate their invasiveness and metastasis (4, 5). CAFs retain their properties *in vitro* without contact with carcinoma cells (6); whether they maintain the activated state due to epigenetic changes has not yet been determined.

Induction of DNA damage in fibroblasts by irradiation increases their ability to aid tumor development (7). Genotoxic agents used for cancer therapy have been shown to induce Wnt16 in fibroblasts, which – in a paracrine manner – activates Wnt signaling in tumor cells and thus attenuates the efficacy of cytotoxic chemotherapy (8). Similarly, senescent fibroblasts, which are characterized by a proinflammatory phenotype and persistent DNA damage, have potent tumor-promoting activity (9–12). Notably, while senescent fibroblasts have no effect on normal epithelial cells, they enhance proliferation of malignant and premalignant epithelial cells (11).

In contrast to CAFs, normal fibroblasts are required to maintain tissue homeostasis and they have been shown to control abnormal growth of pre-neoplastic cells and to restrain tumor progression. For example, inactivation of the TGFβ type II receptor in fibroblasts was sufficient to initiate prostate intraepithelial neoplasia (PIN), confirming that normal fibroblasts can block tumor initiation (5, 13). However, the mechanisms whereby normal fibroblasts inhibit tumor initiation/progression remain unknown.

Tumor cells communicate with the stroma through soluble factors such as cytokines, chemokines, and growth factors. A number of cytokines mediate their responses through activation of the JAK/STAT pathway. STATs (STAT1–STAT6) are latent cytoplasmic transcription factors that transduce signals to the nucleus where they activate transcription, and thereby regulate the expression of a variety of target genes. STAT1 is the founding member of the STAT family. IFNγ was the first cytokine shown to activate STAT1 signaling (14, 15) and STAT1 has been shown to mediate the anti-proliferative activity of IFNs (16). We and others have shown that lack of STAT1 expression perturbs the induction of p21 in response to 5-Aza-CdR (17), to inhibitors of HDAC activity (18), and to camptothecin (Klampfer et al., unpublished). p21 is an inhibitor of cell cycle progression and its regulatory region harbors multiple STAT1 binding sites, suggesting that STAT1 supports transcriptional activation of p21 in response to these agents (19). STAT1 has been suggested to have tumor-suppressor properties (20) and expression of STAT1 suppressed the tumorigenicity of RAD-105 cells *in vivo*, which correlated with decreased expression of proangiogenic molecules such as bFGF, MMP-2, and MMP-9 (21). Although the role of STAT1 in tumorigenesis appears to be complex, these findings demonstrate that STAT1 can act as an inhibitor of tumor progression by restraining tumor growth and metastasis.

In this study, we compared the ability of normal intestinal fibroblasts and myofibroblasts isolated from Crohn’s disease (CD), ulcerative colitis (UC), or colon cancer patients, to regulate the growth of colon cancer cells. We show that normal fibroblasts inhibit proliferation of colon cancer cells and that myofibroblasts isolated from a CD patient, which fail to induce STAT1 signaling in tumor cells, lack this inhibitory activity. We demonstrated that activation of fibroblasts with TNF, a cytokine with a central role in the pathogenesis CD, is sufficient to reduce their ability to induce STAT1 signaling in tumor cells. Fibroblasts failed to inhibit growth of STAT1-deficient tumor cells, confirming a crucial role of STAT1 for the crosstalk between tumor cells and fibroblasts.

## Materials and Methods

### Cell lines and co-culture experiments

The HCT116 and Hke-3 colorectal carcinoma cell lines, which differ only by the presence of the mutant K-Ras allele (22), were cultured in MEM containing 10% FBS. Rat intestinal epithelial cells (IEC-6), transfected with an inducible K-Ras Val-12 cDNA (IEC-iK-Ras), were a generous gift from Dr. Raymond DuBois (23). Cells were maintained in Dulbecco’s modified Eagle’s medium containing 10% fetal bovine serum, 400 μg/ml G418 (Invitrogen), and 150 μg/ml hygromycin B (Sigma). Expression of oncogenic Ras was induced by 5 mM IPTG (Calbiochem). MIC216 cells, a kind gift of Michele Dr. Kedinger (Universite Louis Pasteur, Paris), were maintained in DMEM medium with 10% FBS and human intestinal 18Co fibroblasts (ATCC) in MEM supplemented with 10% FBS. HIF myofibroblasts isolated from a normal donor (HIF ND) and myofibroblasts isolated from a CD patient and an UC patient were a generous gift from Dr. Claudio Fiocchi, The Cleveland Clinic Foundation (24–26). Conditioned media were prepared from sub-confluent fibroblast cultures maintained in MEM containing 10% FBS, centrifuged to remove cell debris and were used immediately or were stored at −80°C. Supernatants collected from CAF were provided by Dr. Mahida, University Hospital Nottingham, UK.

Transwell permeable supports with 0.4 μM pores (Corning Incorporated, Lowell, MA, USA) were used in co-culture experiments, which were performed as described before (27, 28). Briefly, HCT116 colon cancer cells were co-cultured with fibroblasts in a 1:1 ratio for 24 h. Human TNFα and IFNγ were purchased from Biosource Life Technologies. Cells were treated with TNF (5 ng/ml) or IFNγ (5 ng/ml) for 24 h. Phosphorylation of STAT1 (STAT1–Y701) was determined 1 h after treatment with IFNγ (Figure 2C).

Cell growth was assessed by the MTT assay, by BrdU incorporation (BrdU cell proliferation Assay kit, Calbiochem, Gibbstown, NJ, USA) or by CellTiter-Glo Luminescent assay (Promega). The assays were performed according to the manufacturer’s instructions. For all assays, 1 × 10^4^ cells/100 μl were plated into 96 well plates alone or in the presence of fibroblast conditioned medium (CM) as indicated. For the MTT assay, 10 μl of MTT reagent (5 mg/ml) was added to cells for 4 h, and cells were lysed overnight with 0.01 N HCL in 10% SDS. Absorbance wad read at 570 nm, using 630 nm as a reference. For the BrdU incorporation assay, cells were labeled with BrdU for 8 h, fixed and incubated with an anti-BrdU antibody (1:100) for 1 h at room temperature. After 30 min incubation with HRP-labeled antibody, substrate was added for 15 min and absorbance was read at dual wavelength (450–540 nm). The CellTiter-Glo reagent was added to equal volume of the cell culture medium and cultures were mixed for 2 min on an orbital shaker to induce cell lysis. After further incubation for 10 min, luminescence was measured using the plate reader. In all cases, experiments were performed in triplicate.

### Transient transfection and reporter gene assay

HCT116 and Hke-3 cells were transiently transfected using the calcium phosphate method (Promega). The plasmids used in the manuscript were described before. 8XGAS-LUC and 3XAP1-LUC plasmids were provided by Dr. Christopher Glass and the TOP-FLASH promoter construct by Dr. Vogelstein. In all cases, cells were transfected with 1 μg of the test plasmid DNA and 0.1 μg of TK-renilla to control for transfection efficiency and viability. Transfected cells were cultured alone or together with fibroblasts for 24 h. The luciferase and renilla activity were determined according to the vendor’s protocol (Dual Luciferase reporter assay, Promega, Madison, WI, USA).

HCT116 cells were transfected with a pool of four siRNAs (Dharmacon) specific for the human STAT1 gene using the Profection Mammalian Transfection Systems (Promega, Madison, WI, USA) as we described before (18, 29, 30). Non-specific (NSP), non-targeting siRNA, was used as control. Both STAT1 and NSP siRNAs were delivered at a concentration of 25 nM.

### Western blotting

Immunoblotting was performed using standard procedures. Membranes were blocked with 5% milk in TBS containing 0.1% Tween 20, and incubated with antibodies specific for cyclin D1 (Santa Cruz Biotechnology Inc., Santa Cruz, CA, USA), pSTAT1, COX2, IRF1 (Millipore, Billerica, MA, USA), and β-actin (Sigma Aldrich, St. Louis, MO, USA). Immunoreactive bands were visualized by chemiluminescence (Amersham ECL™ western blotting detection kit, Piscataway, NJ, USA).

### Proteome profiler, human cytokine antibody array

The levels of cytokines were determined in supernatants collected from untreated 18Co cells or from 18Co cells that were treated with TNF (5 ng/ml) for 24 h. Relative cytokines levels were determined using Proteome Profiler, Human Cytokine Array kit (R&D Systems, Minneapolis, MN, USA) according to the manufacturer’s instructions. Supernatants were used immediately or were stored at −80°C and were incubated with human cytokine array panel antibody cocktail at room temperature for 1 h. After blocking, the membranes were incubated with supernatants at 4°C overnight on a rocking platform. Membranes were incubated with streptavidin–HRP for 30 min at room temperature and developed with Chemi Reagent Mix according to the manufacturer’s instructions.

### Generation of 3D spheroids by hanging drop cell culture

HCT116 cells were trypsinized and resuspended in a complete MEM medium at a concentration of 1 × 10^6^ cells/ml. Ten microliters of the cell suspension were deposited on the lid of the 100 mm tissue culture dish, which was subsequently inverted onto the PBS-filled bottom chamber. Cells were incubated at 37°C and 5% CO_2_ for 5–7 days and the formation of spheroids was monitored on a daily basis.

## Results

### Intestinal fibroblasts induce STAT1 signaling in colon cancer cells

**Figure 1:**
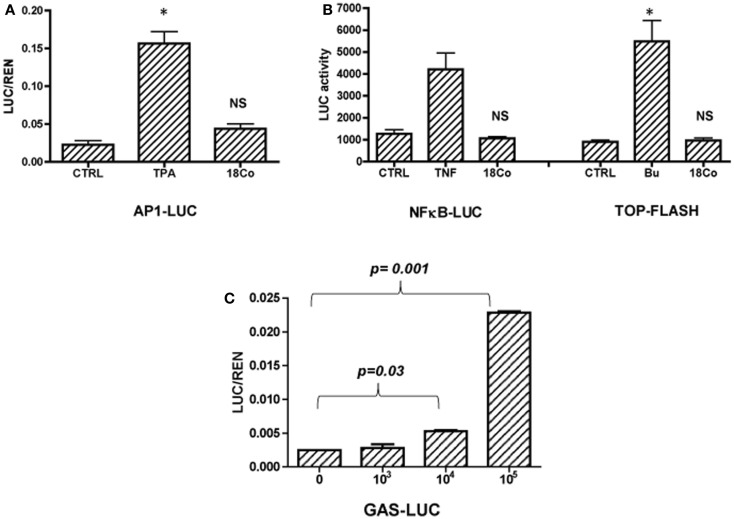
**18Co intestinal fibroblasts induce STAT1 signaling in carcinoma cells**. HCT116 cells were transfected with reported genes for AP1 **(A)**, NFκB, Wnt (TOP-FLASH) **(B)**, or STAT1 (GAS, gamma activated sequence) and were cultured alone or in the presence of 18Co cells. **(C)** HCT116 cells transfected with GAS-LUC were cultured in the presence of increasing number of 18Co cells as indicated. *p = < 0.01 versus control cells; NS, non-significant.

While the protumorigenic nature of CAFs is well studied, little is known about the nature of the crosstalk between tumor cells and normal, tumor-inhibitory stroma. To establish the mechanism whereby normal intestinal fibroblasts regulate the growth of colon cancer cells, we transfected HCT116 cells with reporter genes measuring the activity of major signaling pathways, including AP1, NFκB, Wnt, and STAT1. Transfected cells were cultured in the absence or the presence of 18Co intestinal fibroblasts (1 × 10^5^/well of a 12 well plate) for 24 h. Treatment of cells with 12-*O*-tetradecanoylphorbol-13-acetate (TPA, 10 ng/ml) served as a positive control for AP1-driven transcriptional activity, treatment with TNFα (5 ng/ml) for NFκB and with sodium butyrate (Bu, 5 mM) for the Wnt-driven transcriptional activity. 18Co cells did not impact the activity of NFκB, Wnt, AP1-driven signaling pathways (Figures 1A and 1B), however, we showed that fibroblasts – in a dose-dependent manner – enhanced STAT1-driven transcriptional activation in tumor cells (Figure 1C). Activation of STAT1 transcriptional activity by 18Co cells was cell-type specific, as we published before that macrophages failed to modulate STAT1 transcriptional activity in colon cancer cells (data not shown) (27).

**Figure 2:**
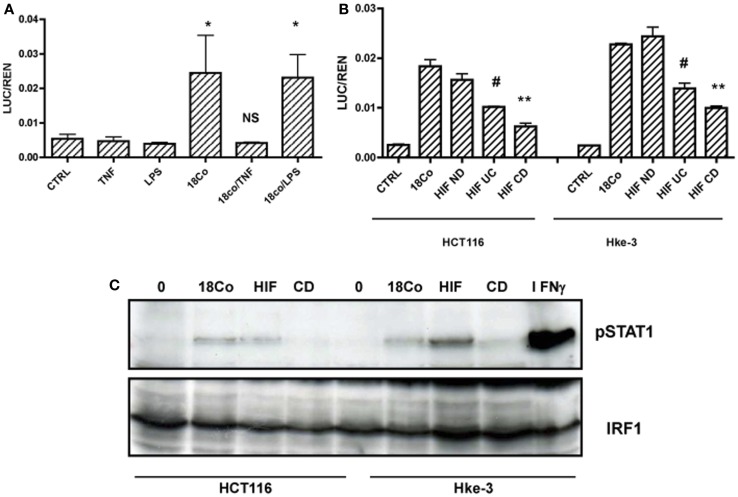
**Fibroblasts induce STAT1 signaling in tumor cells**. **(A)** HCT116 cells transfected with GAS-LUC were treated as indicated, **(B)** HCT116 and Hke-3 cells were transfected with GAS-LUC and were co-cultured with 18Co cells, fibroblasts isolated from a normal donor (HIF ND), a patient with ulcerative colitis (HIF UC) or Crohn’s disease (CD). **(C)** HCT116 and Hke-3 cells were co-cultured with 18Co, HIF, or CD myofibroblasts and the levels of pSTAT1 and IRF1 were determined by immunoblotting. *p = < 0.05 versus control cells; NS, non-significant; ^#^*p* < 0.02; **p < 0.01 versus 18Co and HIF ND fibroblasts.

In contrast to normal fibroblasts, activated fibroblasts produce a number of soluble factors. For example, myofibroblasts isolated from CD patients produce increased levels of TNF, which acts in an autocrine and paracrine manner and plays a major role in the pathogenesis of CD (31, 32). First, we tested whether activation of 18Co cells with TNF, or with LPS, a known inducer of inflammatory mediators, alters their ability to activate STAT1 activity and their crosstalk with tumor cells. HCT116 cells were transfected with the GAS-LUC reporter gene and were cultured alone or together with 18Co cells in the absence or the presence of TNF (5 ng/ml) or LPS (10 ng/ml) for 24 h. Cells were co-transfected with TK-renilla to monitor for transfection efficiency and cell viability, and the results were expressed as the ratio between luciferase and renilla (LUC/REN). As shown in Figure 2A, treatment with TNF, but not LPS, completely prevented the ability of 18Co cells to trigger STAT1 activation in HCT116 cells.

To exclude the possibility that activation of STAT1 signaling was specific for 18Co cells, we used another line of normal intestinal fibroblast. We compared the ability of human intestinal fibroblasts isolated from normal mucosa (HIF ND) and myofibroblasts isolated from a patient with UC or CD, risk factors for colon cancer, to activate STAT1 in colonic epithelial cells. Like 18Co cells, normal HIFs induced STAT1 activation in both HCT116 and Hke-3 cells. The ability of myofibroblasts isolated from UC or CD patients to activate STAT1 signaling in tumor cells was significantly reduced compared to 18Co and HIF ND fibroblasts (Figure 2B).

Consistent with the results shown in Figure 2B, HCT116 and Hke-3 cells that were co-cultured with normal fibroblasts, but not myofibroblasts isolated from a CD patient, showed increased levels of pSTAT1 (Y701), confirming that fibroblast-derived factor(s) induce STAT1 activation in tumor cells (Figure 2C). HCT116 cells treated with IFNγ for 1 h served as a positive control for STAT1 phosphorylation.

**Figure 3:**
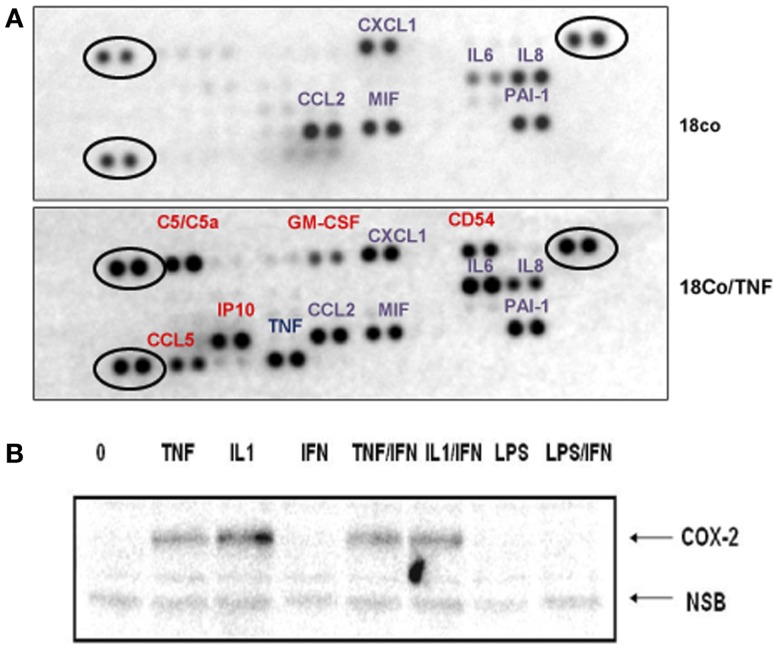
**Activation of 18Co cells with TNF stimulates the synthesis of a number of protumorigenic factors**. **(A)** 18Co cells were left untreated or were treated with TNF for 24 h and the proteome profiler antibody array (R&D Systems) was performed. Circled are positive controls. **(B)** 18Co cells were stimulated with TNF, IL1, IFNγ, or LPS as indicated and the amount of COX2 was determined by immunoblotting.

18Co cells produce several cytokines/chemokines (Figure 3A) that could, in a paracrine manner, contribute to STAT1 activation in intestinal cells. Treatment of 18Co cells with TNF induced the synthesis of a number of protumorigenic soluble factors, including sICAM1 (CD54), C5a, CCL5, GM-CSF, and IP10 (Figure 3A). The expression of IL6 significantly increased and, surprisingly, the expression of IL8 decreased upon TNF treatment. Whether any of these factors interfere with the STAT1 activity remains to be determined. More comprehensive analysis of soluble factors will be required to determine how fibroblast-derived factors regulate the growth of tumor cells and how TNF alters the signaling between tumor cells and fibroblasts. For example, we showed that both TNF and IL1, another proinflammatory cytokine, are potent inducers of COX2 expression in 18Co cells (Figure 3B), which could, by stimulating prostaglandin synthesis, further impact the crosstalk between tumor cells and stroma.

### Normal intestinal fibroblasts, but not myofibroblasts isolated from a Crohn’s disease patient, inhibit the growth of colon cancer cells

**Figure 4:**
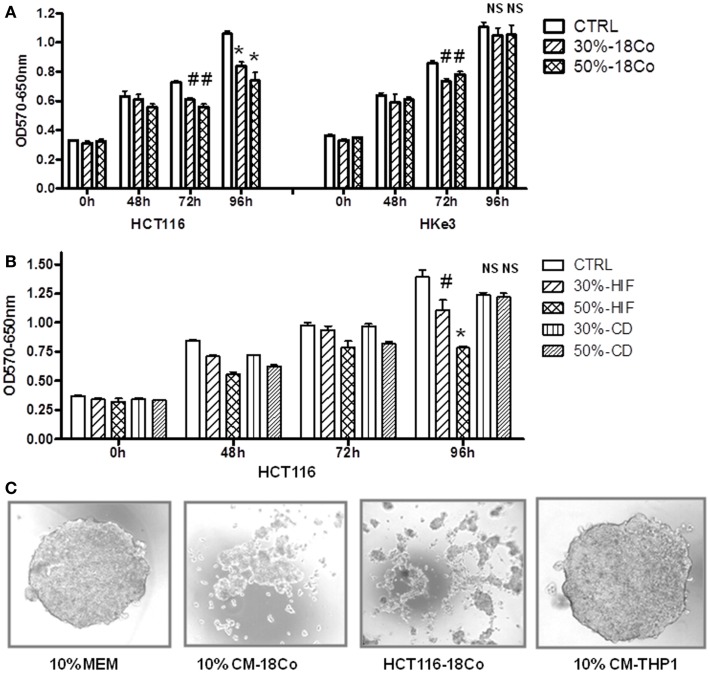
**Normal fibroblasts, but not myofibroblasts isolated from a CD patient restrain the growth of cancer cells**. **(A,B)** HCT116 and Hke-3 cells were cultured alone or in the presence of 30 or 50% of conditioned medium from 18Co cells, intestinal myofibroblasts from a normal donor (HIF), or from a patient with CD as indicated. MTT assay was performed as described in the Section “Materials and Methods.” **(C)** Spheroids of HCT116 cells were generated using a hanging drop technique. Spheroids were generated using control medium (10% MEM), medium containing 10% conditioned medium from 18Co cells (10% CM-18Co) or THP1 macrophages (10% CM-THP1). Alternatively, spheroids were generated by combining HCT116 and 18Co cells (HCT116–18Co). ^#^*p* < 0.05, *p < 0.005, compared to control cells; NS, non-significant.

Next, we examined how fibroblasts impact the growth of human colon cancer cells. Furthermore, to establish whether oncogenic transformation of intestinal epithelial cells with activated K-Ras alters their crosstalk with fibroblasts, we performed experiments in both HCT116 and Hke-3 cells. HCT116 cells harbor a mutant K-Ras V12 allele, and Hke-3 cells are an isogenic derivative, generated by the targeted deletion of the dominantly acting mutant K-Ras allele in HCT116 cells (22). Cancer cells were grown in the presence of control medium or CM from normal intestinal fibroblasts (18Co cells), and the viability of tumor cells was monitored by the MTT assay. As shown in Figure 4A, 18Co cells secrete factors that, in a dose-dependent manner inhibited the growth of colon cancer cells. We confirmed that HIFs, another line of normal human intestinal fibroblasts also inhibited the growth of HCT116 cells (Figure 4B). The results were confirmed by the BrdU incorporation assay (data not shown). In marked contrast, myofibroblasts isolated from a CD patient, which failed to activate STAT1 signaling in tumor cells (Figure 2), had lost the ability to inhibit the growth of tumor cells (Figure 4B).

Significantly, the growth-inhibitory effect of both 18Co and HIF fibroblasts was more pronounced in HCT116 cells, which carry mutant K-Ras, than in HKe-3 cells (Figure 4A, data not shown). Similar results were obtained using rat intestinal epithelial cells with inducible K-Ras expression (Figure S1 in Supplementary Material).

In contrast to the majority of established cell lines, the HCT116 cell line shows limited heterogeneity and has been proposed to consist of mostly stem cells (33). Indeed, HCT116 cells readily form spheroids, confirming their stem-cell phenotype. We showed that soluble factors produced by 18Co cells diminished the ability of HCT116 cells to form spheroids (Figure 4C). Similarly, if we generated spheroids by co-culture of HCT116 and 18Co cells, the size of spheroids was significantly reduced and we did not observe large, compact spheroids, as typically formed by HCT116 cells. This confirmed the negative effect of fibroblast-derived factors on carcinoma cells and suggested that fibroblast-derived factors regulate the stemness of carcinoma cells. In contrast, macrophage-derived factors, which induce Wnt signaling and promote the growth of HCT116 cells (27, 28), did not interfere with the spherogenic potential of HCT116 cells (Figure 4C).

**Figure 5:**
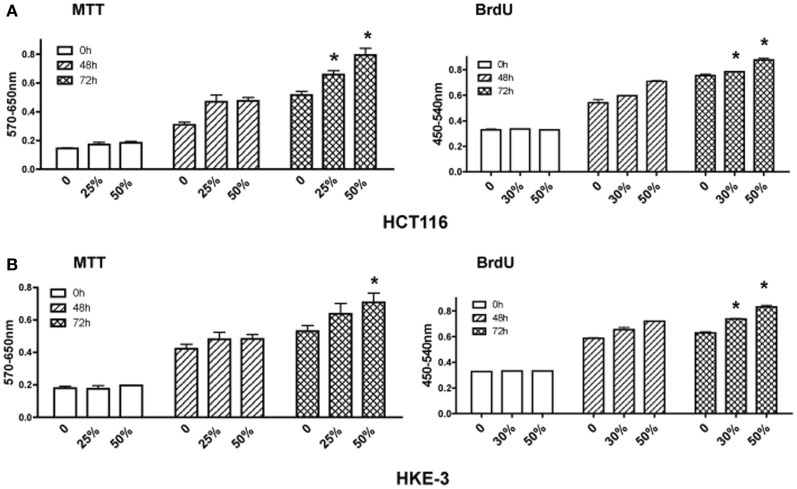
**Cancer-associated myofibroblasts promote growth of tumor cells**. HCT116 **(A)** and Hke-3 cells **(B)** were cultured in the absence or the presence of 25, 30, or 50% of condition medium from human cancer-associated fibroblasts. MTT assay and BrdU incorporation were performed as described in the Section “Materials and Methods” at 48 and 72 h of incubation. *p < 0.05, compared to control cells.

Finally, we investigated how CAFs regulate the growth of HCT116 and Hke-3 cells. Tumor cells were grown in the absence or the presence of CM from cancer-associated myofibroblasts (kindly provided by Dr. Mehida, University Hospital Nottingham, UK) and the growth and proliferation of the cancer cells was monitored by the MTT assay and BrdU incorporation. In contrast to normal myofibroblasts, which inhibited proliferation of cancer cells, we confirmed that factors produced by CAFs promoted growth of both HCT116 and Hke-3 cells (Figure 5), consistent with their protumorigenic activity.

### Fibroblasts inhibit growth of colon cancer cells in a STAT1-dependent manner

**Figure 6:**
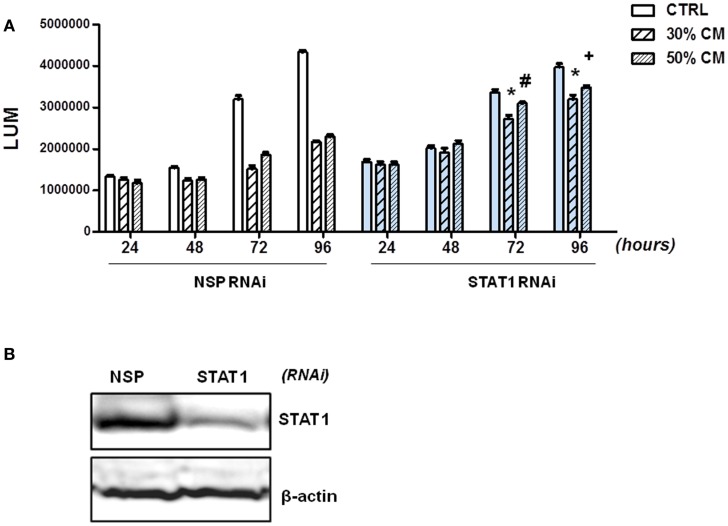
**Fibroblasts inhibit growth of HCT116 cells in a STAT1-dependent manner**. **(A)** HCT116 cells were transfected with non-specific (NSP) RNAi or with STAT1-specific RNAi and cell growth was monitored in the absence (CTRL) or the presence of 30% or 50% conditioned medium (CM) from 18Co cells by CellTiter-GLO Assay (*Promega*) at 24, 48, 72, and 96 h as indicated. **(B)** Silencing of STAT1 in HCT116 cells was confirmed by immunoblotting. *p < 0.0001, ^#^*p* = 0.03, ^+^*p* = 0.0001, compared to cells transfected with NSP RNAi.

To demonstrate that factors produced by normal fibroblasts regulate the growth of tumor cells through STAT1 activation, we silenced STAT1 in HCT116 cells. Efficiency of STAT1 silencing was confirmed by immunoblotting (Figure 6B); STAT1 deficiency did not have a significant impact on the growth of HCT116 cells. Next, we compared the ability of 18Co fibroblasts to modulate the growth of HCT116 cells that were transfected with NSP or STAT1 specific RNAi. As shown before (Figure 4), fibroblasts-derived factors inhibited proliferation of HCT116 cells transfected with NSP RNAi. However, the ability of fibroblasts to inhibit growth was significantly diminished in HCT116 cells transfected with STAT1 RNAi (Figure 6A). While fibroblast-derived factor reduced the viability of HCT116 cells transfected with non-targeted RNAi to 45–50%, HCT116 cells transfected with STAT1-specific RNAi retained 80–89% viability in the presence of fibroblast-derived factors (Figure 4). These results confirmed that fibroblasts restrain the growth of tumor cells, at least in part, through activation of STAT1.

## Discussion

Although tumor progression is commonly driven by the accumulation of somatic mutations, it has been established that tumor cells become addicted not only to oncogenes, but also to supportive signals they receive from the tumor microenvironment. While fibroblasts are the predominant cell type in the lamina propria of normal colon, they are replaced by myofibroblasts in hyperplastic and neoplastic polyps, confirming a role of myofibroblasts in colorectal neoplasia. Carcinoma cells recruit normal fibroblasts and secrete factors that promote their conversion into myofibroblasts. Among tumor-derived factors that activate fibroblasts are TGFβ, PGE, TNF, IFNγ, and IL6. In turn, activated fibroblasts secrete HGF, CCL7, UPAR, VEGF, and other factors that promote stemness, growth, survival, and invasiveness of tumor cells (34, 35). CAFs isolated from invasive tumors share similarities with myofibroblasts, such as high expression of αSMA and increased collagen contractility.

In contrast, normal stromal cells have been shown to produce factors that inhibit the progression of epithelial malignancies (36,37,38) and it has been proposed that a normal tissue microenvironment serves as a barrier to cancer (38). Indeed, normal stroma appears to impede tumor development. However, much less is known about signaling pathways and factors from the normal stromal microenvironment that have the ability to ameliorate or even reverse tumor progression.

In this study, we show that normal fibroblasts induce STAT1 signaling and restrain the growth of colon cancer cells. Genome-wide expression analysis revealed that fibroblasts induce the expression of STAT1 and interferon-responsive genes also in breast cancer cells, suggesting that interaction of breast cancer cells and fibroblasts induces an interferon response (39). Surprisingly, however, in that report the authors found that breast cancer patients with high expression of STAT1 had worse prognosis. We demonstrated that changes in the microenvironment, as they occur in inflammatory bowel disease alter the crosstalk between tumor cells and fibroblasts and result in the inability of myofibroblasts isolated from CD or UC patients to maintain the homeostasis of the intestinal epithelium. We showed that myofibroblasts isolated from CD or UC patients, who are at increased risk of developing colon cancer, were not able to induce STAT1 in carcinoma cells and failed to inhibit their growth. Activation of 18Co cells with TNF, a cytokine that plays a major role in the pathology of CD, was sufficient to prevent STAT1 activation in carcinoma cells. Finally, we confirmed that CAFs promote the growth of colon cancer cells, in accord with their protumorigenic activity. Thus, our data suggest an evolution of the tumor-associated stroma, in which an early step is loss of the ability to restrain the growth of the adjacent epithelium, followed by a gain of protumorigenic function.

We show that intestinal fibroblasts inhibit the growth of carcinoma cells (Figure 4), but have no significant effect on adhesion of HCT116 cells on fibronectin, or on their migration (data not shown). Our data suggest that K-Ras-transformed cells are more sensitive to growth-inhibitory effects of fibroblast-derived factors, although the induction of STAT1 signaling by fibroblasts was not significantly affected by the presence of mutant K-Ras (Figure 2). This is in contrast to our finding that IFN-induced STAT1 activity is impaired in cells with oncogenic K-Ras (18, 40) and suggests that in addition to STAT1 signaling, other pathways are likely to contribute to the restrained growth of Ras-transformed cells in the presence of fibroblasts. We showed that fibroblasts secrete a number of soluble factors, but it is currently unknown which of these factors is responsible for the activation of STAT1 in tumor cells. CCL2, CXCL1, and IL6, produced by 18Co cells (Figure 3A) could contribute to increased STAT1 activity in tumor cells.

We demonstrated that TNF inhibits the ability of fibroblasts to induce growth-inhibitory STAT1 signaling in intestinal cells, potentially contributing to the loss of growth-inhibitory activity of fibroblasts isolated from CD or UC patients. TNF, which has been shown to activate intestinal fibroblasts (41, 42), plays a major role in the pathogenesis of CD and inhibitors of TNF have potent therapeutic activity (43). We show that TNF stimulates the expression of a number of cytokines and chemokines, including GM-CSF, IP10, CCL5, and IL6, but surprisingly it reduces the synthesis of IL8 in 18Co cells (Figure 3A). Another consequence of TNF stimulation is the expression of COX2 in 18Co cells (Figure 3B), which is part of the proinflammatory signature of CAFs (44). Although the mechanism whereby stimulation of fibroblasts with TNF interferes with STAT1 activation in tumor cells remains unknown, PGE2 has been shown to interfere with STAT1 activity (45), and may therefore contribute to the inability of TNF stimulated fibroblasts to activate STAT1 signaling in tumor cells. TNF has also been shown to inhibit IFNα-activated STAT1 and inhibit IFNα signaling in the liver (46). It is likely that several factors contribute to differential effects of normal fibroblasts and CD myofibroblasts on the growth of intestinal cells. For example, myofibroblasts isolated from patients with UC displayed significantly lower expression of SFRP1, a Wnt inhibitor, which has been shown to inhibit proliferation of IEC cells (47).

Because of their pivotal role in tumor progression, CAFs have emerged as an important therapeutic target. They are genetically stable and normalization of the tumor microenvironment is an important venue to pursue in cancer chemoprevention and cancer treatment.

## Conflict of Interest Statement

The authors declare that the research was conducted in the absence of any commercial or financial relationships that could be construed as a potential conflict of interest.

## Supplementary Material

The Supplementary Material for this article can be found online at http://www.frontiersin.org/Journal/10.3389/fonc.2014.00088/abstract

Figure S1 **Fibroblast-derived factors inhibit growth of rat intestinal cells**. Rat IEC-iKRasV12 cells were left untreated or were induced by IPTG to express mutant K-Ras. The cells were cultured in the absence (CTRL) or the presence of 30 or 50% of conditioned medium from rat MIC216 intestinal fibroblasts. Cell growth was monitored by the MTT assay **(A)** or BrdU incorporation **(B)**. The inhibitory effect of MIC216 cells was confirmed by co-culturing of IEC-iKRasV12 and MIC216 cells using transwells **(C)** and by demonstrating the reduced levels of cyclin D1 (cyclD1) in intestinal cells grown in the presence of MIC216 fibroblasts **(D)**. NSB, non-specific band.
