# Supplementary figures and images for: The Role of STAT1 for Crosstalk between Fibroblasts and Colon Cancer Cells
Source: Front Oncol. 2014 Apr 30;4:88. doi: 10.3389/fonc.2014.00088 (PMC4012204; doi:10.3389/fonc.2014.00088)

**A**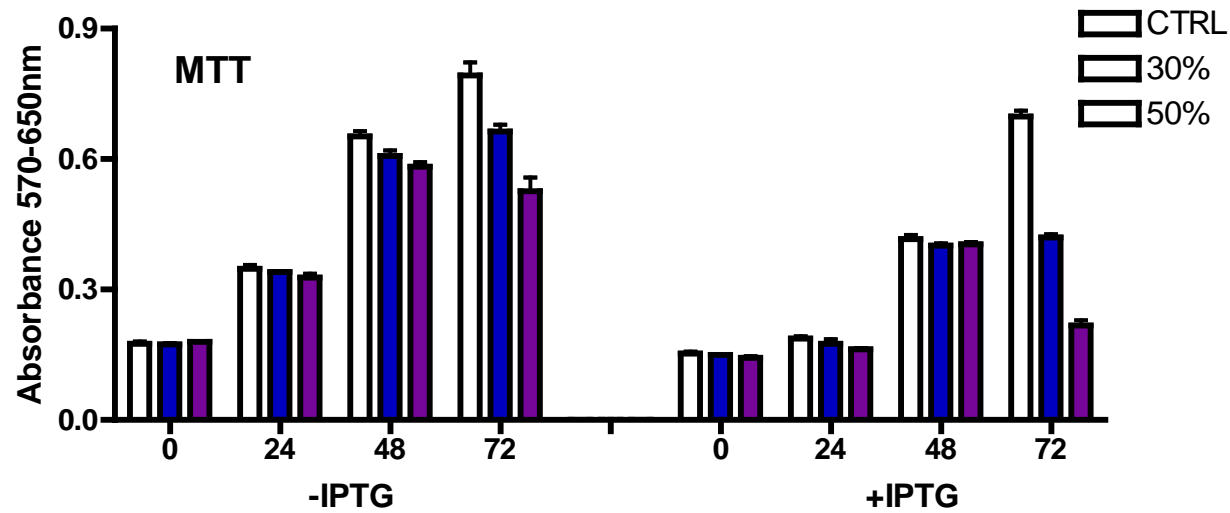**B**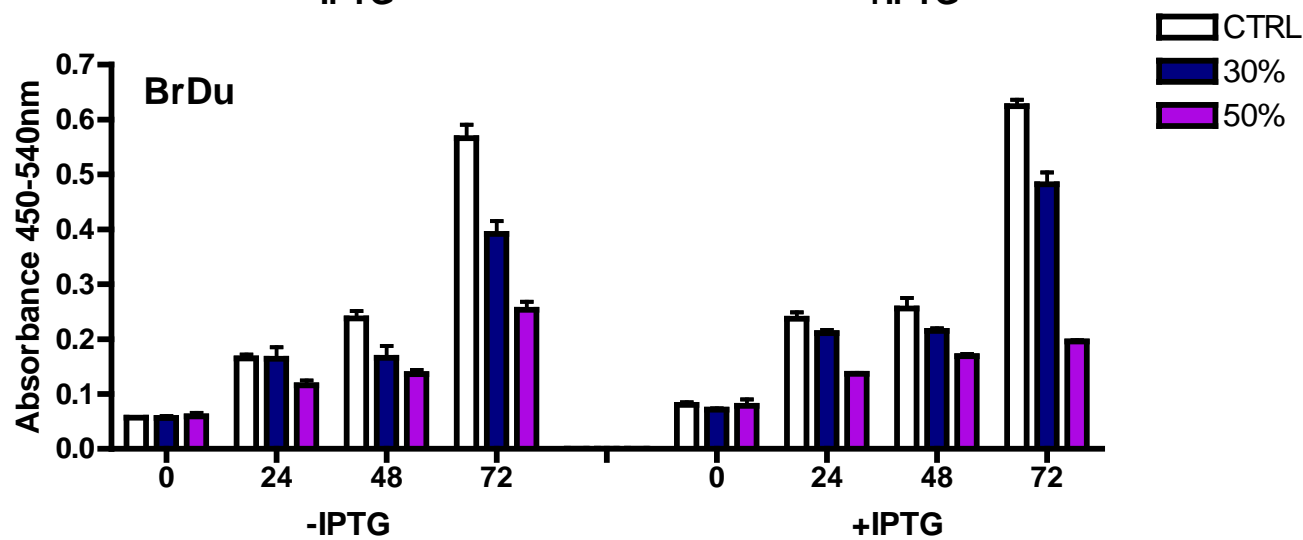**C**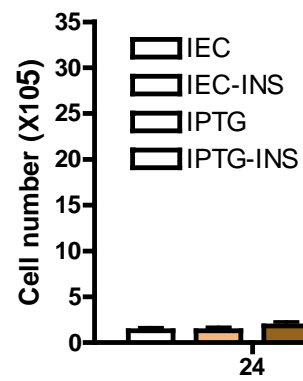**D**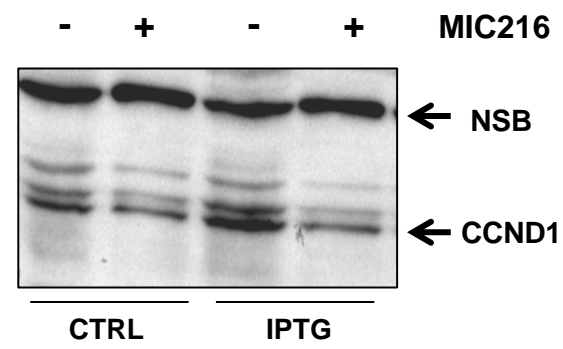

Supplement: Figure S1 — Fibroblast-derived factors inhibit growth of rat intestinal cells. Rat IEC-iKRasV12 cells were left untreated or were induced by IPTG to express mutant K-Ras. The cells were cultured in the absence (CTRL) or the presence of 30 or 50% of conditioned medium from rat MIC216 intestinal fibroblasts. Cell growth was monitored by the MTT assay (A) or BrdU incorporation (B). The inhibitory effect of MIC216 cells was confirmed by co-culturing of IEC-iKRasV12 and MIC216 cells using transwells (C) and by demonstrating the reduced levels of cyclin D1 (cyclD1) in intestinal cells grown in the presence of MIC216 fibroblasts (D). NSB, non-specific band. [file Presentation1.PDF]
